# Transparency in the Ophthalmology Residency Match: Background, Study, and Implications

**DOI:** 10.7759/cureus.19826

**Published:** 2021-11-23

**Authors:** Jonathan C Markle, Harris Ahmed, Kishan Pandya, Ankur Parikh, Youstina Bolok, Jared Fehlman, Varun Aitharaju, Riley Bastian, Shreya Dey, Meghana Chalasani, Meghana Chanamolu, Karina Pedersen, Natalie Ganios, Vincent Pham, Shabnam Mansur, Janice C Law

**Affiliations:** 1 School of Medicine, Northeast Ohio Medical University, Rootstown, USA; 2 Department of Ophthalmology, Loma Linda University Medical Center, Loma Linda, USA; 3 Department of Ophthalmology, University of Cincinnati Medical Center, Cincinnati, USA; 4 Department of Ophthalmology, Vanderbilt University Medical Center, Nashville, USA

**Keywords:** san francisco match, ophthalmology match, ophthalmology residency, residency filters, residency transparency, residency match

## Abstract

Background

Medical students are applying to dramatically more ophthalmology residency programs than in the past, causing an increased administrative burden for programs and financial harm to students. This study considers the background of this situation and looks at how a lack of transparency surrounding potential residency match filters contributes. Furthermore, this study raises several potential solutions to this lack of transparency that may increase the functionality of the ophthalmology residency match.

Objective

The purpose of this study was to determine the availability and consistency of potential ophthalmology residency match filters through training program websites and the American Medical Association’s (AMA) Residency & Fellowship Database (FREIDA).

Methods

This study was a cross-sectional observational study of ophthalmology residency program websites and AMA's FREIDA database entries. For 119 ophthalmology residency programs, five potential filters were evaluated for both availability and consistency on individual residency websites and FREIDA. These filters were: (1) whether a program required a minimum United States Medical Licensing Examination (USMLE) Step 1 score; (2) minimum number of letters of recommendation required; 3) whether a minimum USMLE Step 2 score was required; (4) if the program accepts the Comprehensive Osteopathic Medical Licensing Examination (COMLEX) sequence in lieu of the USMLE; and (5) ability of the residency to sponsor a visa (J-1, H-1B, or F-1). Each program's website and FREIDA entry were independently evaluated by two authors to increase validity, with a third author brought in to break the tie in case of a disagreement.

Results

Only two ophthalmology residency programs had information about all five filters both available and consistent on their website and FREIDA. Inter-reviewer reliability was 92.5%.

Conclusions

Information about potential filters used in the ophthalmology residency match is neither publicly available nor consistent. This lack of transparency may contribute to the phenomenon of medical students applying to dramatically more ophthalmology residency programs. A standardized database of these filters is needed to increase transparency to applicants, which may reduce the expenses of medical students and the workload of program directors.

## Introduction

The mean number of applications submitted by applicants to ophthalmology residencies has increased dramatically over the last decade, from 52 in 2011 to 79 in 2021 [1,2]. This trend is not unique to ophthalmology, even though the specialty is described as a competitive match by the American Academy of Ophthalmology [3-7]. This increase in applications has caused an increased administrative burden for residency programs and financial harm to students. The cost of submitting applications through San Francisco (SF) Match is $60 for the first 10 programs, $10 each for programs 11-20, $15 each for programs 21-30, $20 each for programs 31-40, and $35 for each program beyond 40. Medical students in the 2021 match cycle applied to an average of 79 programs, totaling $1,875 if a medical student applies to this number [8].

When considering the medical student perspective, it is easy to foresee that this trend will continue despite increased penalties to apply to more programs. The financial consequences of not matching are far more devastating than these fees: a year of attending physician salary lost, plus the additional accrued interest from student loans. For students with families, the toll is even greater, as they must figure out how to support their loved ones without loan income for the next year. Some students who wish to try to match again in ophthalmology undertake a pre-residency research fellowship to improve their curriculum vitae, which requires an additional move and is often unpaid [9-11]. The match rate for re-applicants to ophthalmology is significantly lower (51% match rate for allopathic graduates compared to 85% for allopathic seniors; 33% for osteopathic graduates compared to 55% for osteopathic seniors) [12]. Medical educators have noted that this situation is a prisoner’s dilemma, where medical students must apply to ever-greater numbers of programs to maximize their chances of matching [13,14].

Medical students are also aware that residency programs use filters that can screen out their application if they do not meet certain “non-negotiables” (e.g., a minimum United States Medical Licensing Examination [USMLE] Step 1 score) [15-17]. However, programs often do not inform students which filters are used, let alone whether their application will be perceived as competitive if it makes it past the filters. Without access to these two critical pieces of information, it is much more difficult to create a targeted list of programs to apply to. Furthermore, this lack of informational access disadvantages students without home departments and mentors in their desired specialty by obfuscating information about how they should spend their extracurricular time. Students without “insider access” to home ophthalmology departments and mentors may not be able to acquire information about these cutoffs independently. Only 53% of US medical schools are associated with an ophthalmology residency, and only 58% are associated with an ophthalmology department [18]. Predictably, the match rate for schools with an associated ophthalmology residency is 1.4x higher than those without [19].

Increasing transparency in residency selection criteria has been put forward as a potential solution to the increasing application numbers [20-22]. To this end, the National Resident Matching Program (NRMP) publishes a biennial report called the NRMP Program Director (PD) survey. PD surveys in each specialty represented in the NRMP match are queried about what selection factors are important for obtaining an interview and being ranked highly on the final match list. The three most commonly cited factors for obtaining an interview are the USMLE Step 1 score, letters of recommendation (LORs) in the specialty, and the USMLE Step 2 clinical knowledge (CK) score, all of which are potential non-negotiables [23]. The Association of American Medical Colleges (AAMC) has also created a database called Residency Explorer, which conglomerates information about individual NRMP residencies into one database and allows users to gauge their competitiveness by comparing their application profile to matched applicants at each program [24]. However, since ophthalmology uses the SF Match, which neither participates in the PD survey nor shares data with Residency Explorer, information about selection criteria for ophthalmology residency is far less accessible.

Without tools like the PD survey and Residency Explorer to identify what factors an individual ophthalmology residency values, medical students must rely on program websites and third-party databases to gauge whether or not their applications will be reviewed, let alone be competitive for matching. The most widely known of these databases is the American Medical Association’s (AMA) Residency & Fellowship Database (FREIDA), which charges $20 for a one-year medical student membership [25]. Residency programs that fill out expanded listings on FREIDA provide some information as to what filters they use, as well as some statistics on current residents. This allows estimations of whether an applicant will pass the program’s filters as well as their competitiveness. Another widely used database is Texas STAR (Seeking Transparency in Application to Residency), which is a student-reported application database, and the student participation rate is below 50% [26]. While this allows insight as to the average characteristics of some students who interviewed and/or matched at a given residency, it gives no information about which application components are truly “non-negotiable” and which are not. Furthermore, the validity of Texas STAR may be limited, as not every US medical school participates in its annual survey [26].

The purpose of this study was to assess the availability and accuracy of information about potential “non-negotiables” (e.g., filters) that applicants to ophthalmology residency must meet for their application to be fully considered.

## Materials and methods

A total of 119 US ophthalmology residency programs were identified using the 2020 SF Match Resources for Medical Students website [27]. This website, updated yearly by the SF Match, gives medical students access to a list of the participating programs in the year's ophthalmology residency match.

Links to websites of all 119 identified programs were cataloged in a spreadsheet. The programs' corresponding entries in the AMA FREIDA database were then identified, with all 119 found. Each year, FREIDA contacts all Accreditation Council for Graduate Medical Education (ACGME)-sponsored residencies and asks if they would like to voluntarily provide information about the residency to the database. This information may include hospital locations, application requirements (i.e., filters), demographics, resident benefits, work schedule, and call schedule. However, individual programs may choose to provide all, some, or none of this information to FREIDA.

Information about five potential filters was evaluated for availability and consistency on the individual ophthalmology program websites and corresponding FREIDA entries. The five filters were: (1) Does the program requires a minimum USMLE Step 1 score? (2) Does the program requires a minimum USMLE Step 2 CK score? (3) What is the minimum number of LORs the program requires? (4) Does the program accepts the Comprehensive Osteopathic Medical Licensing Examination (COMLEX) sequence in lieu of USMLE scores? (5) Could the program accept applications from international medical students (e.g., sponsors J-1, H-1B, or F-1 visas)?

Minimum USMLE Step 1, USMLE Step 2 CK, and LORs were analyzed due to their station as the three most commonly cited factors for obtaining an interview in NRMP programs. Acceptance of the COMLEX sequence in lieu of USMLE was evaluated because more than 40% of Doctorate of Osteopathic Medicine (DO) students do not take the USMLE sequence [28]. Finally, acceptance of international students was evaluated as the limited literature on this subject suggests it is the most used match filter [29,30].

**Figure 1:**
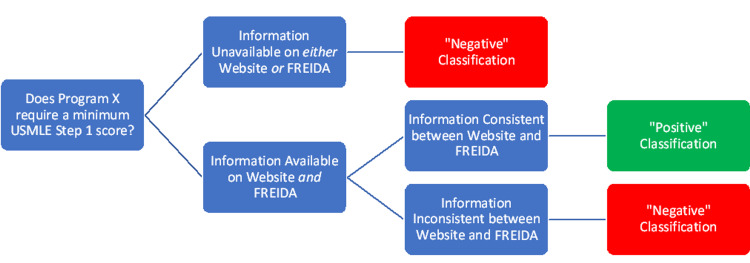
Example decision tree for classifying a program as "positive" or "negative." FREIDA, Residency & Fellowship Database; USMLE, United States Medical Licensing Examination.

Consistency of information between residency websites and FREIDA was evaluated if the information was available in both places. If the information for each criterion was available and consistent between the program website and FREIDA, the result was classified as “positive.” Otherwise, the result was classified as “negative” (Figure 1).

For each of the five criteria, the percentage of programs that had information both available and consistent across both sources was calculated. The number out of five criteria that were available and consistent for each program was also calculated. Each residency program was independently evaluated by two authors to increase validity. In case of a disagreement between the two reviewers, a third reviewer was brought in to break the tie.

## Results

The number of ophthalmology residency programs that had information about all five potential “non-negotiables” both available and consistent on their websites and FREIDA was 2 (2%). The mean number of filters available and consistent across all programs was less than 1 (mean = 0.29). Each filter was then considered separately to determine which ones were more likely to be available (Tables 1, 2).

**Table 1:** Number of ophthalmology residencies stating they did or did not use a particular filter on their website (n = 119). LOR, letter of recommendation; COMLEX, Comprehensive Osteopathic Medical Licensing Examination.

Filter	Count (%)
Step 1	16 (13%)
LOR	19 (16%)
Step 2	10 (8%)
COMLEX	6 (5%)
Visa sponsorship	40 (34%)

**Table 2:** Number of ophthalmology residencies stating they did or did not use a particular filter on FREIDA (n = 119). LOR, letter of recommendation; COMLEX, Comprehensive Osteopathic Medical Licensing Examination; FREIDA, Residency & Fellowship Database.

Filter	Count (%)
Step 1	37 (31%)
LOR	38 (32%)
Step 2	35 (29%)
COMLEX	41 (34%)
Visa sponsorship	42 (35%)

Inter-reviewer reliability was calculated by counting every instance of disagreement between two reviewers about the availability of a given residency filter. For each residency, there were 10 areas of agreement or disagreement (see Tables 1, 2). The overall inter-rater agreement was 92.5%.

## Discussion

The results of this study point to a need for a central, standardized database of “non-negotiables” from each program. As the USMLE Step 1 exam transitions to pass/fail scoring in 2022, this need will become even more pressing. Recent surveys of PD across specialties, including ophthalmology, indicate that greater emphasis will be placed upon Step 2 CK scores and the prestige of the applicant’s medical school, which may disadvantage students who do not take Step 2 CK by the time they apply to the SF Match, as well as students from less prestigious medical schools and under-represented minorities [31,32]. To mitigate the various impacts of the COVID-19 pandemic on the 2020-2021 residency match cycle, Quillen et al. emphasized the need for increased transparency of residency programs [33]. They describe the Association of University Professors of Ophthalmology (AUPO) initiative to collect and publish 12 ophthalmology residency program data points for every program such as average resident cataract volume, number of full-time faculty, and association with a veteran’s hospital training site. While these are important and often easily searchable metrics, none of the 12 data points include the five filters from this study [33].

Not only do students need easy access to accurate training program information through the 12 AUPO data points, but by including the five filters from this study, students could benefit from learning about a program’s preferences, giving students guidance on how to best spend their time and money. Otherwise, the differential match rates between medical students with and without an associated ophthalmology program and a student’s access to mentorship may persist or worsen. This lack of informational access may also disproportionally hurt students who are underrepresented minorities in medicine (URiM) (Black or African Americans, Hispanics or Latinos, and American Indians or Alaskan Natives). This bears out in the very low percentage of URiM’s who are practicing ophthalmologists (6% compared to 30.7% of the US population) [34]. URiM students are also disproportionally affected by issues of cost in applying to both medical school and residency [35]. Programs posting their “non-negotiables” will decrease cost burdens for these students (and all medical students) by allowing them to specifically target programs they will be considered for, also decreasing the administrative burden for residency directors [35,36].

New standardized metrics may need to be developed; an intriguing option is creating an ophthalmology exam for medical students based on the recommendations of the AUPO Medical Student Education Council. According to the AUPO, every graduating medical student should be able to “(1) describe the anatomy of the eye and the visual system, (2) perform a basic eye examination, (3) evaluate a patient with acute painless vision loss, (4) evaluate a patient with chronic vision loss, (5) evaluate a patient with a red or painful eye, (6) evaluate a patient with eye trauma, (7) evaluate a patient with an eye movement abnormality or diplopia, (8) describe the important causes of vision loss in children, (9) describe the ocular manifestations of systemic disease, (10) list the most important ocular side effects of systemic drugs, (11) list the common ocular medications that can have systemic side effects, and (12) describe when it is necessary to refer a patient urgently to ophthalmology” [37]. Creating an exam to measure these competencies may both increase transparency in what ophthalmology programs value while better preparing medical students for their residency education. However, care would be needed in the creation of such an exam to make sure it would not disadvantage students with less exposure to ophthalmology during medical school. This could be accomplished by the AUPO releasing a standardized set of study materials that covers all exam content or linking to existing study resources that already do so.

This study also has implications for the education of osteopathic students. If a majority of ophthalmology residency programs do not accept the COMLEX in lieu of the USMLE, as the limited data suggests, then perhaps efforts should be made to create a single licensing exam for both allopathic and osteopathic students. As it stands, all osteopathic students must take a licensing exam that most ophthalmology residencies might not accept, wasting both their time and money as they must then take the USMLE sequence to be considered, also furthering the gap for financially disadvantaged students [28,38]. With the Doctorate of Medicine (MD)/DO residency single accreditation completed, efforts to decrease the duplicity between the two training paths at the medical school level must be considered.

The limitations of this study only underscore the need for increased transparency. Only five potential “non-negotiables” were evaluated in this study, but a given program may use more or less. As such, any database created should include information about other potential filters, which may include Alpha Omega Alpha (AOA) membership, leadership and service experience, research activity, whether an away rotation is required, and advanced degrees. There is no guarantee that either FREIDA or individual residency websites are accurate or up to date, emphasizing the need for a centralized and standardized resource in this realm. Finally, as previously mentioned, applicants also need access to information about whether their application will be given fair consideration if they make it through the filters (e.g., are they truly “competitive” in the eyes of a given residency). Studies have been undertaken to identify criteria that make students more competitive applicants for ophthalmology residency. In addition to the aforementioned criteria, being selected for AOA membership, attending a top 25 medical school, attending an allopathic school, and being geographically proximal to a residency program have all been identified as criteria significantly increasing the likelihood of successfully matching [19,39]. However, these studies do not capture the nuance of individual residency programs, only globally assessing the ophthalmology residency landscape.

## Conclusions

Information about potential filters used in the ophthalmology residency match is neither publicly available nor consistent. This lack of informational access may be contributing to the recent increase in the average number of applications medical students send to ophthalmology residencies. A standardized database of these filters is needed to increase transparency to applicants, which may reduce the expenses of medical students and the workload of program directors.
